# p21-activated kinase group II small compound inhibitor GNE-2861 perturbs estrogen receptor alpha signaling and restores tamoxifen-sensitivity in breast cancer cells

**DOI:** 10.18632/oncotarget.6081

**Published:** 2015-11-06

**Authors:** Ting Zhuang, Jian Zhu, Zhilun Li, Julie Lorent, Chunyan Zhao, Karin Dahlman-Wright, Staffan Strömblad

**Affiliations:** ^1^ Department of Biosciences and Nutrition, Novum, Karolinska Institutet, Huddinge, Sweden; ^2^ Department of Oncology and Pathology, Karolinska Institutet, Solna, Sweden; ^3^ Science for Life Laboratory (SciLifeLab), Karolinska Institutet, Solna, Sweden

**Keywords:** PAK4, ERα, tamoxifen resistance, phosphorylation, small molecule inhibitor

## Abstract

Estrogen receptor alpha (ERα) is highly expressed in most breast cancers. Consequently, ERα modulators, such as tamoxifen, are successful in breast cancer treatment, although tamoxifen resistance is commonly observed. While tamoxifen resistance may be caused by altered ERα signaling, the molecular mechanisms regulating ERα signaling and tamoxifen resistance are not entirely clear. Here, we found that PAK4 expression was consistently correlated to poor patient outcome in endocrine treated and tamoxifen-only treated breast cancer patients. Importantly, while PAK4 overexpression promoted tamoxifen resistance in MCF-7 human breast cancer cells, pharmacological treatment with a group II PAK (PAK4, 5, 6) inhibitor, GNE-2861, sensitized tamoxifen resistant MCF-7/LCC2 breast cancer cells to tamoxifen. Mechanistically, we identified a regulatory positive feedback loop, where ERα bound to the PAK4 gene, thereby promoting PAK4 expression, while PAK4 in turn stabilized the ERα protein, activated ERα transcriptional activity and ERα target gene expression. Further, PAK4 phosphorylated ERα-Ser305, a phosphorylation event needed for the PAK4 activation of ERα-dependent transcription. In conclusion, PAK4 may be a suitable target for perturbing ERα signaling and tamoxifen resistance in breast cancer patients.

## INTRODUCTION

Breast cancer is the most common cancer worldwide and the second most frequent cancer mortality in females [1]. 70% of all breast cancers are estrogen receptor α (ERα) positive, where ERα constitutes a driving force for breast cancer progression [2]. ERα exerts its functions through binding to estrogen responsive elements (EREs) or by indirect binding to DNA through other transcription factors, which subsequently induces the expression of target genes [3]. ERα positive breast cancer patients often benefit from ERα antagonist treatment. As such, tamoxifen is the most commonly used ERα antagonist in the clinic. Although tamoxifen largely improves breast cancer patient survival, the development of tamoxifen-resistance is common. Several mechanisms could contribute to tamoxifen resistance, including alterations in estrogen signaling and/or crosstalk between estrogen signaling and growth factor signaling pathways [4, 5]. In particular, post-translational modifications of ERα, such as phosphorylations, may play important roles in regulating estrogen signaling thereby overcoming tamoxifen responsiveness [6].

The PAK (p21-activated kinase) family are serine/threonine kinases acting downstream of the small GTPases Cdc42 and Rac with regulatory roles of cytoskeletal dynamics [7]. PAKs are classified into Group I (PAK1-3) and Group II (PAK4-6) based on sequence homology, although all PAKs have an N-terminal GTPase (Rac/Cdc42)-binding regulatory domain and a C-terminal kinase domain [8]. Interestingly, PAKs may play functional roles in several oncogenic events, including oncogenic transformation, invasion and metastasis [8]. However, the different PAK family kinases may exert different or even opposite effects. In fact, this appears to be the case in ERα signaling, where PAK1 and PAK6 may regulate ERα signaling in opposite directions. While immunohistochemical staining of PAK1 in breast cancer patient specimens was correlated to tamoxifen resistance [9, 10], possibly related to PAK1-mediated phosphorylation of ERα [11–14], PAK6 binds ERα and inhibits its transcriptional activity, and this PAK6-ERα interaction could be enhanced by tamoxifen [15]. Nevertheless, the possible mechanistic involvement of PAK kinases in tamoxifen resistance remains unclear.

Interestingly, a number of findings suggest that PAK4 may be involved in cancer progression [16]. For example, PAK4 is up-regulated in most human cancer cell lines [17], and has also been found to be overexpressed in patient material of several human cancer forms, including colon, esophageal, pancreas, ovarian cancer, and breast cancer [18–21]. Importantly, high PAK4 expression in ovary cancer was correlated with progressing disease stage, poor patient survival and to resistance to chemotherapy [19]. Functionally, PAK4 may play a role in transformation, since dominant-negative PAK4 partially inhibited Ras-induced transformation in NIH3T3 mouse embryonic fibroblasts; a constitutively active PAK4 mutant transformed NIH3T3 cells *in vitro* [17, 22]; and overexpression of either activated or wild-type PAK4 made NIH3T3 cells tumorigenic in athymic mice *in vivo* [21]. PAK4 may also be required for anchorage-independent growth of NIH3T3 cells and HCT116 human colon carcinoma cells [17, 22]. In breast cancer cells, PAK4 inhibits cell adhesion [22–24] and promotes cell migration by selectively inducing αvβ5 mediated breast cancer cell motility through the phosphorylation of the integrin β5 cytoplasmic tail and by regulating actin depolymerisation through phosphorylation of LIMK1 [24–28]. Moreover, PAK4 may also protect mouse fibroblasts and HeLa cells from apoptosis by phosphorylating BAD, a potentially tumor promoting effect [29]. However, the potential role of PAK4 in breast cancer remains largely elusive, for example whether PAK4 may directly affect breast cancer related proteins such as ERα.

At the same time, there is an urgent need to find ways to overcome tamoxifen resistance in the clinic, including the identification of targets affecting the tamoxifen response. To this end, the recent development of different PAK inhibitors now facilitates the testing of their suitability for treatment of breast cancer [30–32]. However, it is unclear which PAKs may be suitable targets to overcome tamoxifen resistance.

Here, using two distinct gene expression databases, each containing information from more than 1900 breast cancer patients, we found that high PAK4 expression consistently correlated with poor outcome for endocrine treatment and specifically tamoxifen treated breast cancer patients, while the expression of other PAK family members did not consistently display such correlation in both databases. Mechanistically, we discovered a novel positive feedback loop between ERα and PAK4, where ERα binds to the PAK4 gene and induces the expression of PAK4, and where PAK4 in turn phosphorylates ERα, promotes ERα protein stability and its transcriptional activity. Importantly, while overexpression of PAK4 caused decreased sensitivity to tamoxifen in MCF7 human breast cancer cells, a specific inhibitor of group II PAKs, GNE-2861, restored the sensitivity to tamoxifen in the tamoxifen-resistant MCF-7/LCC2 cells. Taken together, PAK4 appears as an interesting target to explore for the restoration of tamoxifen sensitivity in breast cancer.

## RESULTS

### Association between PAK4 gene expression and clinical outcome among tamoxifen treated breast cancer patients

**Figure 1:**
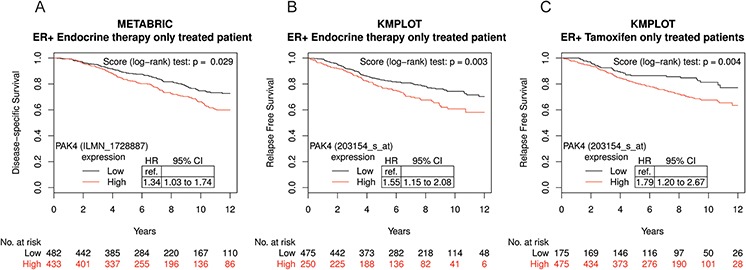
High PAK4 mRNA expression levels correlate with poor survival of endocrine-treated breast cancer patients **A.** Kaplan-Meier plot of disease-specific survival in ER+, endocrine therapy only treated patients in the Metabric database stratified for high (red) and low (black) PAK4 expression levels (*n* = 915; median cut-off; Probe ILMN_1728887: H*R* = 1.34; 95% CI: 1.03–1.74; *P* = 0.029). **B.** Kaplan-Meier plot showing that high PAK4 expression correlates with relapse free survival in ER+, endocrine therapy only treated patients in the KMplot database stratified for high (red) and low (black) PAK4 expression levels (*n* = 725; optimized cut-off; Probe 203154_s_at: H*R* = 1.55; 95% CI: 1.15–2.08; *P* = 0.003). **C.** Kaplan-Meier plot showing that high PAK4 expression correlates with relapse free survival in ER+, tamoxifen-only treated patients in the KMplot database stratified for high (red) and low (black) PAK4 expression levels (*n* = 650; optimized cut-off; Probe 203154_s_at: H*R* = 1.79; 95% CI: 1.20–2.67; *P* = 0.004).

The potential prognostic role of PAK family members in endocrine therapy-treated patients was explored in two large public breast cancer datasets, Metabric and KMplot [33, 34], where we assessed two related end-points, disease-free survival and relapse-free survival, respectively, since identical endpoints were not available. We found that high PAK4 expression was associated with poor disease-specific survival among the 915 Metabric ERα positive endocrine therapy-treated patients in a univariable model (Figure 1A, HR = 1.34; 95% CI: 1.03–1.74). In the Metabric database, while endocrine treated patients can be identified, no information is available in terms of the specific endocrine treatment. However, in the KMplot breast cancer database, we were able to analyze a tamoxifen-only treated patient group. In the selected KMplot populations of 725 ERα positive patients treated only with endocrine therapy as systemic adjuvant treatment (“endocrine therapy only”), high PAK4 expression was also associated with poor prognosis (Figure 1B, HR = 1.55; 95% CI: 1.15–2.08). Most patients in the endocrine treated group received tamoxifen. Importantly, also among cases with tamoxifen as the only systemic adjuvant treatment (“tamoxifen-only”) (*n* = 650), high PAK4 expression was correlated to poor relapse-free survival (Figure 1C, HR = 1.79; 95% CI: 1.20–2.67) in a univariable model for relapse-free survival. The patient outcome in tamoxifen-only treated group in relation to PAK4 expression was consistent with that of the endocrine treated patients in both the Metabric and the KMplot datasets. However, while high PAK2 and PAK6 expression correlated with relapse-free survival in the KMplot dataset, no such correlation was detectable in the Metabric dataset (Supplementary Figure S1 and S2). Thus, no consistent correlation was detected between the expression of PAK family members other than PAK4 (PAK1, 2, 3, 5, 6) and the patient outcome of endocrine treated breast cancer patients in the two databases.

The correlation between high PAK4 expression and unfavorable endocrine treated breast cancer patient outcome is consistent with a potential role for PAK4 in tamoxifen resistance. This motivated us to further examine such a potential role for PAK4.

### Pharmacological targeting of group II PAKs restores tamoxifen sensititvity in human breast cancer cells

**Figure 2:**
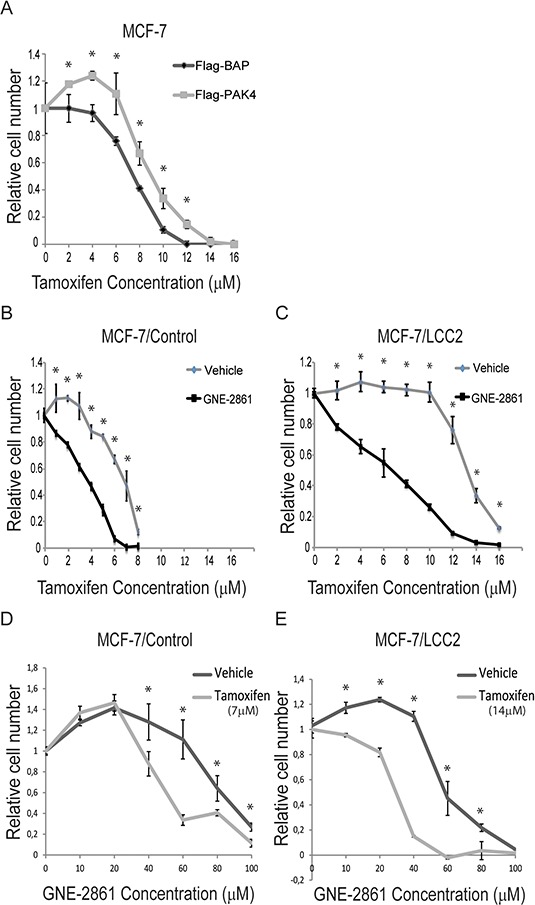
The group II PAK inhibitor GNE-2861 restores tamoxifen-sensitivity in breast cancer cells **A.** PAK4 overexpression inhibits the tamoxifen-response in MCF-7 ERα positive human breast cancer cells. MCF-7 cells with stable transfection of Flag-PAK4 or Flag-BAP control were treated with the indicated tamoxifen concentrations for 48 h and the number of cells was quantified by a WST-1 assay. Shown values represent mean ± s.d. (n = 3) for each concentration, representative for three independent experiments. * - *P* < 0.05 compared to control, according to *t*-test. **B–C.** The group II PAK inhibitor GNE-2861 sensitizes breast cancer cells to tamoxifen treatment. B) MCF-7/Control cells and C) MCF-7/LCC2 tamoxifen resistant cells were treated with vehicle or 50 μM GNE-2861. In addition, each group of cells was treated with the indicated concentrations of tamoxifen for 48 h and the number of cells was quantified by a WST-1 assay. Shown values represent mean ± s.d. (n = 3) for each concentration, representative for three independent experiments. * - *P* < 0.05 compared to control, according to *t*-test. **D–E.** Tamoxifen sensitizes breast cancer cells to GNE-2861 treatment. D) MCF-7/Control cells were treated with vehicle or 7 μM tamoxifen. E) MCF-7/LCC2 tamoxifen resistant cells were treated with vehicle or 14 μM tamoxifen. In addition, each group of cells was treated with the indicated concentrations of GNE-2861 for 48 h and the number of cells was quantified by a WST-1 assay. Shown values represent mean ± s.d. (n = 3) for each concentration, based on three independent experiments. * - *P* < 0.05 compared to control, according to *t*-test.

Based on the prognostic role of PAK4 in endocrine treated and tamoxifen-only treated breast cancer patients, we examined if PAK4 may affect the tamoxifen response in human breast cancer cells. As shown in Figure 2A, stable overexpression of PAK4 significantly decreased the tamoxifen sensitivity in human MCF-7 breast cancer cells (Figure 2A). It can be noted that low concentration of tamoxifen promoted cell proliferation, consistent with the well-established estrogen-like effect of low tamoxifen concentrations [35]. Interestingly, a recently developed small organic compound, GNE-2861 (Compound 17), specifically inhibits group II PAKs (PAK4, 5, 6), but not group I PAKs (PAK1, 2, 3) [31], thereby facilitating pharmacological targeting of group II PAKs in cells. We selected this inhibitor because it displays the most impressive specificity profile among known PAK4-inhibitors [30, 31, 36]. Importantly, GNE-2861 enhanced the tamoxifen sensitivity in MCF-7 cells (Figure 2B), as well as in the tamoxifen-resistant human breast cancer cell line MCF-7/LCC2 [37, 38] where GNE-2861 restored the tamoxifen sensitivity to a similar level as in untreated tamoxifen-sensitive MCF-7 maternal cells (Figure 2B–2C). As shown in Figure 2B–2C, the approximate IC50 of tamoxifen in MCF-7/Control and MCF7/LCC2 cells are 7 μM and 14 μM, respectively. By keeping the tamoxifen concentration constant at the approximate IC50 for each cell line and varying the concentration of GNE-2861, we found that tamoxifen sensitized breast cancer cells to GNE-2861 treatment (Figure 2D–2E). Together, this indicates that PAK4 contributes to tamoxifen resistance in breast cancer cells and may be used as a target to restore tamoxifen sensitivity. Further, this indicates that the group II PAK inhibitor GNE-2861 may be a candidate for the development of tamoxifen-sensitizing pharmacological treatment.

### PAK4 is a direct ERα target gene

**Figure 3:**
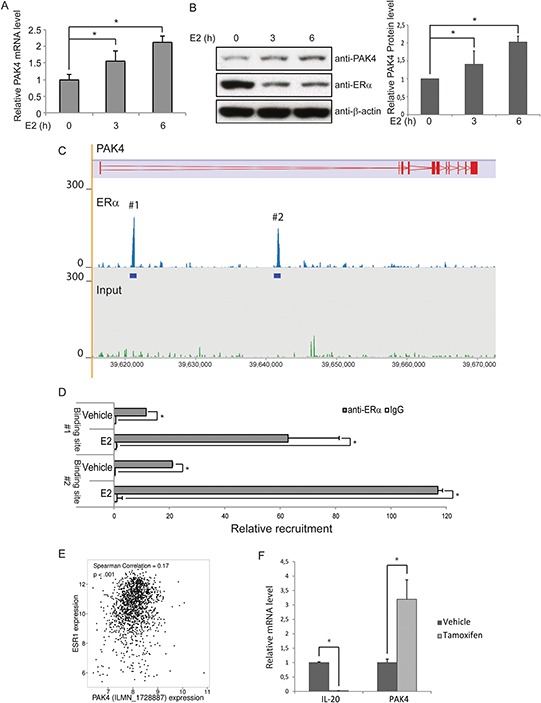
ERα binds to the PAK4 gene and promotes PAK4 expression **A–B.** Induction of PAK4 mRNA and protein by E2. Serum-starved MCF-7 cells were treated with10 nM E2 for up to 6 h. A) The levels of PAK4 mRNA were assessed by qPCR, using 36B4 as an internal control. B) Left: The protein levels were determined by immunoblotting, using β-actin as a loading control. Right: Quantification of PAK4 signal in the immunoblot. Shown values represent mean ± s.d. (n = 3). * - *P* < 0.05 compared to control, according to *t*-test. **C.** ChIP-seq results show the ERα binding peaks within the PAK4 gene locus after E2 treatment. The PAK4 gene is represented in the top track. ERα ChIP-seq signal is shown in the middle track and input ChIP-seq signal in the bottom track. **D.** ChIP-qPCR analysis confirms the recruitment of ERα to the two regions of the PAK4 gene indicated in C. Data presented are normalized to input DNA and expressed as fold enrichment over IgG. Shown values are mean ± s.d. (n = 3). * - *P* < 0.05 as compared with IgG controls, according to *t*-test. **E.** Positive correlation between ESR1 and PAK4 (probe ILMN_1728887) gene expression in ER+ breast cancer patients in the Metabric database (*n* = 1394). Spearman correlatio*n* = 0.17; *P* < 0.001. **F.** Tamoxifen regulation of PAK4 mRNA levels. MCF-7 cells were treated with 1 μM tamoxifen for 12 h. The relative IL-20 (positive control for tamoxifen effect) and PAK4 mRNA levels were assessed by qPCR, using 36B4 as an internal control. Shown values represent mean ± s.d. (n = 3). * - *P* < 0.05 compared to control, according to *t*-test.

The clinical correlation between PAK4 and endocrine treated breast cancer patient outcome together with the functional role of PAK4 in tamoxifen response suggest that there may be a regulatory relationship between PAK4 and ERα. To elucidate this potential relationship, we treated MCF-7 cells with 10 nM 17β-estradiol (E2), a natural ligand of ERα. Interestingly, E2 treatment increased both PAK4 mRNA and protein levels in a time dependent manner (Figure 3A and 3B). Also, our results (Figure 3B) are consistent with the well-known phenomena that E2 treatment significantly reduces ERα protein levels [39]. In addition, analysis of the ERα genomic DNA-binding profile in MCF-7 cells by chromatin immunoprecipitation followed by sequencing (ChIP-seq) revealed two ERα binding sites within intron 1 of the PAK4 gene (Figure 3C). The data have been deposited in NCBI's Gene Expression Omnibus (Zhuang et al., 2015) and are accessible through GEO Series accession number GSE73320 (http://www.ncbi.nlm.nih.gov/geo/query/acc.cgi?acc=GSE73320). These two binding sites were also observed in other published ERα cistromes [40, 41] and one of the binding sites was also found to be present in the ERα cistrome of 1,234 binding sites identified by Lin and colleagues [42]. Chromatin immunoprecipitation followed by quantitative PCR (ChIP-qPCR) validated the binding of ERα to these two regions (Figure 3D), an interaction that was increased by E2 treatment. Together, this indicates a regulatory role for ERα signaling on PAK4 expression via direct ERα binding to the PAK4 gene. Interestingly, analysis of data from ER+ breast cancer patients in the Metabric database shows a positive correlation between ESR1 and PAK4 gene expression (Figure 3E), which is consistent with an ERα regulation of the PAK4 gene in breast cancer.

Interestingly, we found that tamoxifen induced PAK4 mRNA expression in MCF-7 cells (Figure 3F). To avoid off-target effects of tamoxifen, 1 μm tamoxifen was used to treat MCF-7 cells for 12 h. The ERα downstream target gene, IL-20, was used as a positive control for the tamoxifen treatment. The tamoxifen mediated increase in PAK4 expression may allow PAK4 to influence the tamoxifen response.

### PAK4-inhibition decreases ERα protein levels, ERα signaling and E2-mediated cell proliferation

**Figure 4:**
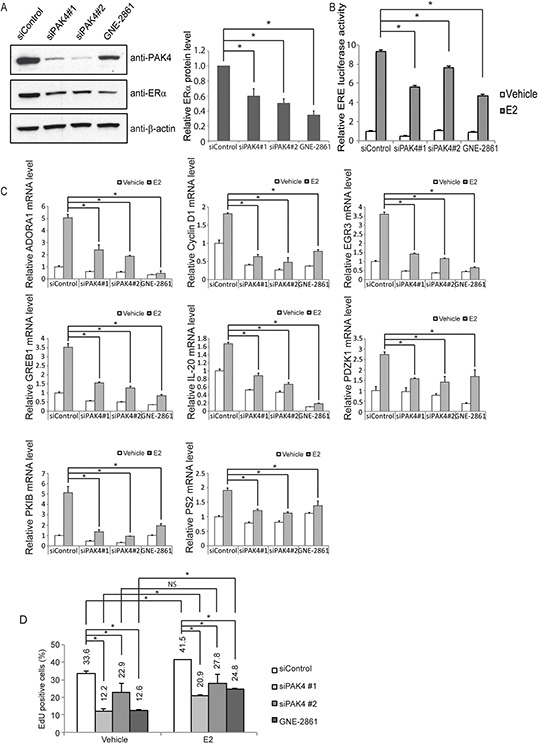
PAK4 inhibition impairs ERα signaling in MCF-7 cells **A.** Left: PAK4 depletion or functional inhibition of group II PAKs reduces ERα protein levels in MCF-7 cells. MCF-7 cells were transfected with siControl or siPAK4 oligos (#1 or #2) for 72 h, or treated with 50 μM GNE-2861 for 24 h. ERα, PAK4 and β-actin levels were determined by immunoblotting. Right: Quantifications of the immunoblot. Shown values represent mean ± s.d. (n = 3). * - *P* < 0.05 compared to control, according to *t*-test. **B.** PAK4 depletion or functional group II PAK inhibition reduces the activity of estrogen receptor-induced signal transduction in MCF-7. MCF-7 cells were transfected with siControl, siPAK4 oligos (#1 or #2), or treated with GNE-2861 as described above. 24 h before measurement, cells were transfected with an ERE luciferase reporter. After 18 h, cells were treated with 10 nM E2 or vehicle, and an ERE-luc luciferase assay was carried out 6 h after E2 addition. Shown values represent mean ± s.d. (n = 3), representative for three independent experiments. * - *P* < 0.05 compared to control, according to *t*-test. **C.** PAK4 depletion or pharmacological group II PAK inhibition decreases the expression of the endogenous ERα target genes ADORA1, Cyclin D1, EGR3, GREB1, IL-20, PDZK1, PKIB, and PS2. MCF-7 cells were transfected with siControl, siPAK4 oligos (#1 or #2), or treated with GNE-2861 as described above. Cells were treated with 10 nM E2 or vehicle for 6 h before harvest and RNA was prepared. The mRNA expression levels of the endogenous ERα target genes were determined by qPCR. Shown are the results from triplicate experiments. Shown values represent mean ± s.d. (n = 3), representative for three independent experiments. * - *P* < 0.05 compared to control, according to *t*-test. **D.** PAK4 depletion or functional group II PAK inhibition impairs cell proliferation in MCF-7 cells. MCF-7 cells were transfected with siControl, siPAK4 oligos (#1 or #2), or treated with GNE-2861 as described above. Cells were then treated with 10 nM E2 or vehicle for 24 h before fixation. EdU was added at a concentration of 10 μM during the last 1 h. The cells were subject to flow cytometry analysis quantifying EdU-positive cells (Supplementary Figure S4). Shown values represent mean ± s.d. (n = 3), which is representative for three independent experiments. * - *P* < 0.05, NS=not significant, as compared to control, according to *t*-test.

While ERα signaling regulated PAK4 expression, we also wanted to find out if PAK4 may regulate ERα signaling. Specifically, in the light of group II PAK-inhibition leading to ERα antagonist (tamoxifen) sensitization, we firstly analyzed the potential of PAK4-inhibition on ERα signaling. To this end, siRNA-mediated knock-down of PAK4 as well as the GNE-2861 compound caused decreased ERα protein levels in both MCF-7 and T47D human breast cancer cell lines (Figure 4A and Supplementary Figure S3A). In order to further elucidate the effect of PAK4 perturbation on ERα transcriptional activity, we performed an estrogen response element (ERE) luciferase assay. Interestingly, PAK4 inhibition by either siRNA or the GNE-2861 compound decreased ERE luciferase activity upon E2 treatment in both MCF-7 and T47D cells (Figure 4B and Supplementary Figure S3B). In line with this, PAK4 inhibition also decreased ERα target gene expression, including ADORA1, Cyclin D1, EGR3, GREB1, IL-20, PDZK1, PKIB, and PS2, both with and without E2 treatment (Figure 4C). Block of PAK4 also significantly decreased EdU incorporation in MCF-7 breast cancer cells both in the presence and absence of E2 stimulation (Figure 4D and Supplementary Figure S4). These results indicate that PAK4 strongly modulates ERα signaling and ERα-regulated cell proliferation.

### PAK4 regulates ERα protein stability

**Figure 5:**
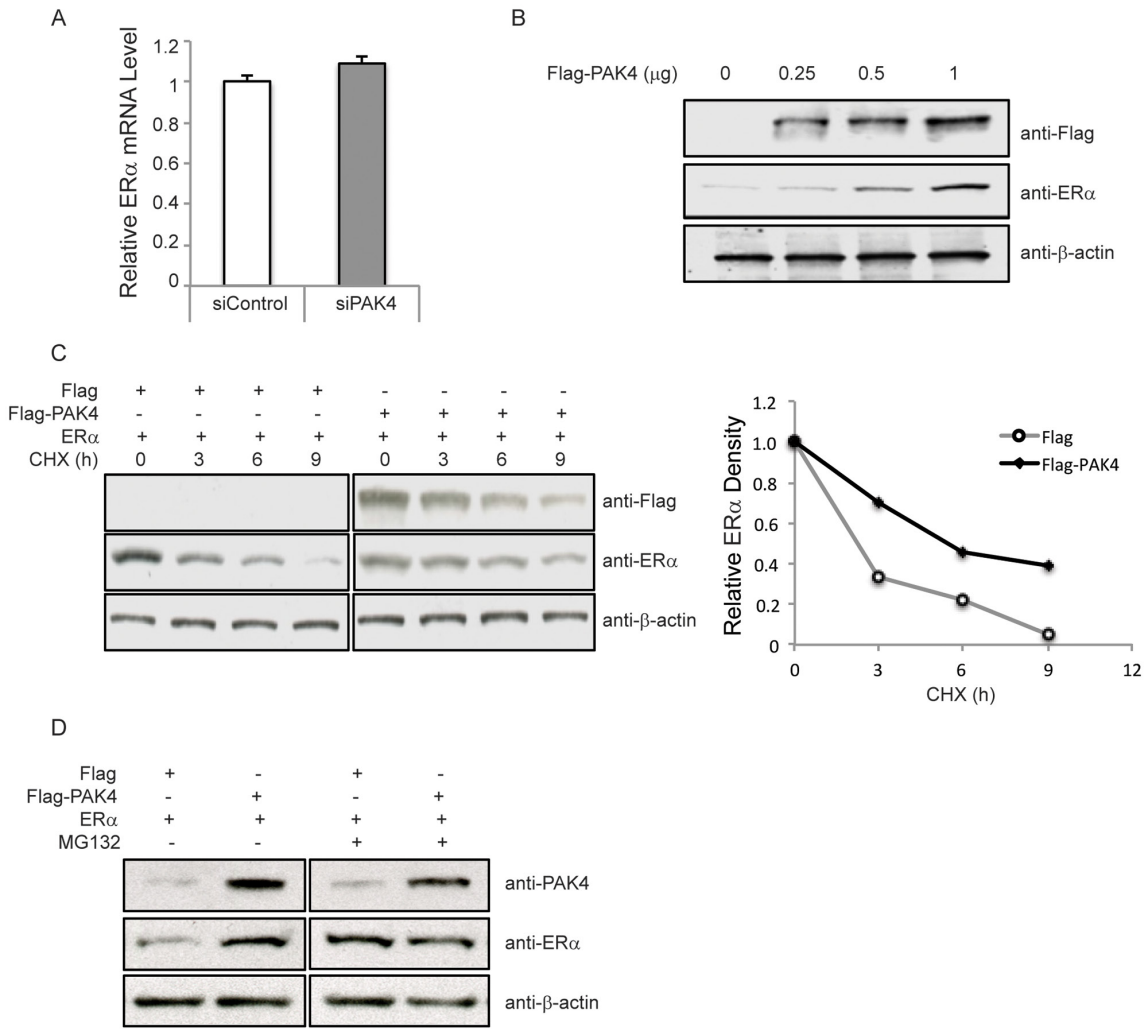
PAK4 regulates ERα protein stability **A.** Depletion of PAK4 does not affect ERα mRNA levels. MCF-7 cells were transfected with control siRNA or PAK4 siRNA (#1 and #2 pool). After 72 h, cells were harvested for analysis. ERα mRNA was measured by qPCR. Shown values represent mean ± s.d. (n = 3). **B.** Over-expression of PAK4 increases ERα protein levels. MCF-7 cells were transfected with varying amounts of a Flag-PAK4 plasmid, and Flag-PAK4, ERα and β-actin levels were determined by immunoblotting. **C.** Over-expression of PAK4 increases ERα protein stability. HEK293 cells were transfected with ERα together with Flag-PAK4 or a Flag control plasmid. Cells were treated with 100 μM cycloheximide (CHX) for the indicated times. Flag-PAK4, ERα and β-actin levels were determined by immunoblotting. The relative ERα protein levels were quantified by ImageJ and normalized to the zero time point ERα levels (before CHX treatment). Two-sample Kolmogorov-Smirnov test (two-sample KS-test) of the curve distributions in three distinct experiments yielded a statistically discernable difference between Flag control and Flag-PAK4 (*P* = 0.03). **D.** The proteasome inhibitor MG132 increases ERα protein level in a similar manner as PAK4 overexpression. HEK293 cells were transfected with ERα together with Flag-PAK4 or a Flag control plasmid. Forty-eight hours after transfection, cells were treated with 10 μM MG132 for 8 h. ERα, PAK4 and β-actin levels were determined by immunoblot analysis. The results are representative for three independent experiments.

As shown in Figure 4A, PAK4 inhibition in MCF-7 cells caused decreased ERα protein levels. Further, ERα mRNA levels were also detected. However, siRNA-mediated PAK4 knock-down did not affect ERα mRNA levels (Figure 5A). Further, transfection of increasing amounts of a PAK4 expression plasmid caused a gradual increase of ERα protein (Figure 5B). ERα protein stability was then analyzed by measuring ERα protein levels at different time points after cycloheximide (CHX) treatment. Importantly, ERα regulates its own mRNA expression in MCF-7 cells, making it difficult to distinguish direct effects of PAK4 on ERα protein levels in this cell line [43]. We therefore performed assays in HEK-293 cells, in which ERα is not expressed. PAK4 overexpression significantly increased exogenous ERα protein half-life in HEK293 cells (Figure 5C). In addition, cells were treated with the proteasome inhibitor MG132, which reduces degradation of ubiquitin-conjugated proteins in mammalian cells. Interestingly, PAK4 overexpression could not further enhance the ERα protein level when ERα proteasomal degradation was inhibited by MG132 (Figure 5D) suggesting that PAK4 overexpression may prevent proteasomal degradation of ERα. Together, our data shows that PAK4 controls ERα signaling via regulation of its protein stability.

### PAK4 regulates ERα signaling through phosphorylation of ERα Serine-305

**Figure 6:**
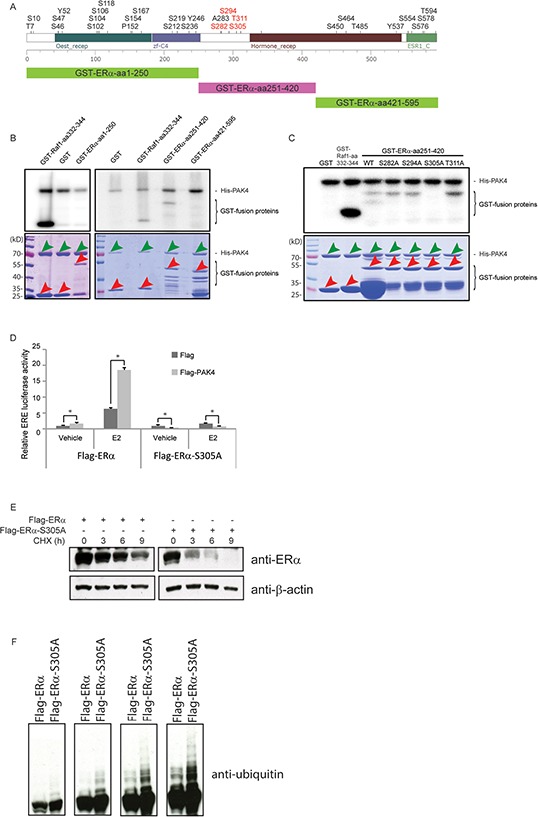
PAK4 regulation of ERα signaling through phosphorylation of ERα-Ser305 **A.** Upper panel: prediction of phosphorylation modification sites of ERα using the PhosphoSitePlus online tool (http://www.phosphosite.org, accessed on May 2014). The four putative Ser/Thr kinase target sites in ERα aa 251–420 are in red. Lower panel: the three different fragments for which we produced GST-fusion proteins used in B. **B.** PAK4 phosphorylates the ERα fragment aa 251–420. His-PAK4 phosphorylation of ERα was analyzed by *in vitro* phosphorylation using recombinant His-PAK4 together with GST-ERα fragments as substrates, using GST alone as a negative control and GST-Raf1-aa332–344 as a positive control (upper panel). The lower panel shows the amounts of His-PAK4 proteins (green arrows) and GST-fusion proteins (red arrows) used in the assay by Coomassie Brilliant Blue gel staining. Bands not demarcated by arrows likely represent degradation products. A size marker is displayed in the left lane of each gel. **C.** PAK4 phosphorylates ERα at Serine 305. Recombinant His-PAK4 was used as the kinase (green arrows in lower panel), and purified GST-ERα aa 251–420 protein fragments with or without mutations (red arrows in lower panel) were substrates. A GST-Raf1-aa332–344 fusion protein was used as a positive control and GST only was used as a negative control (Also red arrows in lower panel). The upper panel is an autoradiography image, the lower panel is a Coomassie Brilliant Blue staining. **D.** ERα-Ser305 phosphorylation is necessary for the PAK4 mediated ERα signaling. Flag control or Flag-PAK4 and wild type Flag-ERα or Flag-ERα-S305A were transfected in the indicated combinations in HEK293 cells. 24 h before measurement, cells were transfected with an ERE luciferase reporter. After 18 h, cells were treated with 10 nM E2 or vehicle, and an ERE-luc luciferase assay was carried out 6 h after E2 addition. Shown values represent mean ± s.d. (n = 3), which is representative for three independent experiments. * - *P* < 0.05 for Flag-PAK4 group versus control, according to *t*-test. **E.** ERα-S305A is less stable than wild-type ERα. HEK293 cells were transfected with wild-type Flag-ERα or Flag-ERα-S305A plasmids. Forty-eight hours after transfection, cells were treated with 100 μM cycloheximide (CHX) for the indicated times. ERα and β-actin levels were determined by immunoblotting. The results are representative for three independent experiments. **F.** ERα-S305A displays stronger ubiquitination than wild-type ERα. HEK293 cells were transfected with wild-type Flag-ERα or Flag-ERα-S305A plasmid. Forty-eight hours after transfection, cells were treated with 10 μM MG132 for 8 h. Ubiquitin were detected by immunoblotting. The panel displays varying exposure times increasing from the left to the right.

Given that PAK4 is a kinase, we examined if PAK4 may directly phosphorylate ERα and thereby affect ERα signaling. First, we expressed and purified fragments of ERα (aa 1–250; aa 251–420; aa 421–595) as GST-fusion proteins (Figure 6A), and performed an *in vitro* kinase assay using purified recombinant PAK4 together with the ERα protein fragments. Interestingly, we found PAK4-mediated phosphorylation of the ERα fragment aa 251–420; while none of the other parts of ERα were phosphorylated (Figure 6B). In order to identify the specific phosphorylation site(s), the possible threonine and serine phosphorylation sites in ERα aa 251–420 were identified according to the PhosphoSitePlus online tool (http://www.phosphosite.org, accessed on May 2014) [44], revealing four such putative Ser/Thr kinase target sites (Figure 6A). Each of these four Ser/Thr was separately mutated into Alanines and tested for the effect of the Ala-mutation in terms of PAK4-mediated phosphorylation. As shown in Figure 6C, while three of these mutations had no effect on PAK4-mediated phosphorylation of ERα aa 251–420; the ERα-S305A mutation abolished PAK4-mediated ERα-phosphorylation. Thus, Ser305 was identified as the PAK4-mediated ERα-phosphorylation site. To test if PAK4-mediated phosphorylation of ERα-Ser305 had any effect on ERα signaling within cells, we examined the ERα response in an ERE luciferase assay in the presence or absence of E2. Importantly, while PAK4 increased the estrogen response by wild type Flag-ERα, the Flag-ERα-S305A mutant failed to respond to PAK4 even in the presence of E2 (Figure 6D). This suggests that PAK4 regulates ERα signaling through the direct phosphorylation of ERα-Ser305. To test the influence of this phosphorylation site for protein stability, wild-type Flag-ERα and Flag-ERα-S305A were overexpressed in HEK293 cells and the stability was determined after CHX treatment. Interestingly, Flag-ERα-S305A displayed a lower stability than wild-type Flag-ERα (Figure 6E). As shown in Figure 6F, Flag-ERα-S305A was also more strongly ubiquitinated than wild-type Flag-ERα, which is consistent with a more repid proteasome-mediated degradation of the Flag-ERα-S305A mutant.

## DISCUSSION

Tamoxifen resistance is a significant clinical problem in breast cancer, causing tumor relapse and progression [45]. However, the mechanism for tamoxifen resistance is not entirely clear, although multiple pathways have been implicated in the presumably diverse mechanisms responsible for tamoxifen resistance, such as estrogen signaling pathways, cell cycle signaling pathways and growth factor receptor pathways [46]. Either loss of ERα function or increased function (or loss of control) can be associated with tamoxifen resistance. The phosphorylation of ERα Ser305 results in activation of ERα, which could render estrogen-hypersensitivity and antiestrogen-insensitivity [13, 47]. In fact, experimental and clinical data suggest that ERα Ser305 phosphorylation may promote tamoxifen resistance in breast cancer [12–14, 47–49]. One possible mechanism for the ERα Ser305 phosphorylation in tamoxifen resistance is an altered orientation between ERα and its coactivator SRC-1, which elevates the ERα transcription activity in the presence of tamoxifen [47, 48]. Moreover, phosphorylation of ERα Ser305 by PAK1 can trigger a secondary phosphorylation on Ser118, which may also contribute to tamoxifen resistance [13]. Our finding that the group II PAK inhibitor GNE-2861, which does not inhibit group I PAKs (PAK1, 2, 3) [31], restored tamoxifen sensitivity, in combination with the induction of tamoxifen resistance by PAK4 overexpression and the correlation of high PAK4 expression with poor patient outcome after tamoxifen treatment now suggest that PAK4 may promote tamoxifen resistance in ERα positive breast cancer. Our identification of PAK4-mediated phosphorylation of ERα-Ser305 is particularly interesting in the light of the previous correlation between ERα-Ser305-phosphorylation and tamoxifen-resistance in breast cancer patients [49]. However, given that ERα Ser305 phosphorylation can be mediated by at least also PAK1, a group I PAK member [49, 50], and by PKA [47, 48, 51], the relative importance of these kinases in breast cancer remains unclear, considering also that both PAK1 and PAK4 have been found overexpressed in human breast cancer [10, 21]. However, while high PAK4 expression displayed consistent correlation with poor outcome of endocrine treated breast cancer patients in two distinct large databases, no other PAK member displayed such a consistent correlation with the patient outcome. However, while the correlation of high PAK4 expression and poor patient outcome is consistent with a functional role in tamoxifen resistance, it should be noted that the PAK kinases are regulated also at the level of kinase activity. Unfortunately, there is at present no good marker for PAK activity that can be traced in patient materials and therefore, it remains unclear if the activity of any of the PAKs may be correlated with disease outcome. Also, in relation to the PAK4 expression level correlation with patient outcome, it should be noted that PAK4 in breast cancer may play other or additional roles to tamoxifen resistance that may influence that patient outcome. Nevertheless, our experimental data indicate that PAK4 may be a suitable pharmacological target for the development of therapy to sensitize breast cancer to tamoxifen treatment.

In addition, we here showed that ERα binds to the PAK4 gene and promotes PAK4 transcription upon E2 treatment, while the increased PAK4 in turn phosphorylates and stabilizes the ERα protein, thereby enhancing ERα signaling and transcription of ERα target genes, including PAK4. This positive feed-forward loop may also promote breast cancer cell proliferation and tamoxifen resistance. So far, PAK4 is the only PAK family member found to be a transcriptional target of ERα. Together, our data indicate that PAK4 may have an unusually tight relationship with ERα signaling.

A related mechanism for tamoxifen resistance is through the loss of expression of Rho guanine dissociation inhibitor α (RhoGDIα), a negative regulator of the Rho family proteins Rho, Rac-1 and Cdc42 [11]. Consequently, loss of RhoGDIα caused increased Rho, Rac-1 and Cdc42 activities in breast cancer cells [11]. Given that PAK1 can act downstream of Rac-1 and Cdc42, Barone et al. proposed that PAK1-mediated phosphorylation of ERα-Ser305 was coupled to the RhoGDIα-regulation of tamoxifen resistance [11]. However, the possible functional involvement of Rho, Rac-1, Cdc42, or PAK1 in the RhoGDIα control of tamoxifen resistance has not been experimentally addressed. In this context, it may be noted that all the six PAK family members are downstream effectors of Rac-1 and Cdc42 [52, 53]. This means that our finding of a role for PAK4 in tamoxifen resistance and in regulating ERα signaling through the phosphorylation of Ser-305 may suggest that PAK4 activation could potentially act down-stream of loss of RhoGDIα in induction of tamoxifen resistance.

Recently, there has been an increasing interest in using PAK family kinases as targets in cancer [8, 16]. To this end, the group I p21-activated kinase inhibitor FRAX1036, in combination with taxane, induced apoptosis in luminal breast cancer cells [32]. Here we showed that GNE-2861, a small molecule selectively inhibiting group II PAKs, but with no effect on group I PAKs [31], overcomes tamoxifen resistance. Given that PAK4 overexpression promoted tamoxifen resistance, and that PAK4 displayed the only consistent correlation in both databases among group II PAKs to breast cancer patient outcome, and that depletion of PAK4 by siRNA gave very similar effects on ERα signaling as GNE-2861, this suggests that GNE-2861 may act to restore tamoxifen sensitivity by the inhibition of PAK4, although we cannot exclude possible effects onto other targets.

## MATERIALS AND METHODS

### Mammalian cell expression constructs

The ERα plasmid and Flag-ERα plasmid was described previously [54]. A Flag-ERα-S305A mutant was generated using the Flag-ERα and the QuickChange Site-Directed Mutagenesis kit (200518-5, Agilent Technologies, Santa Clara, USA). The Flag-PAK4 plasmid was described in previous studies [22, 24].

### Cell culture and transient transfections

DMEM (41965-039, Life Technologies, Grand Island, USA) with 10% fetal bovine serum (FBS) was used to culture MCF-7 (purchased from ATCC), MCF-7/Control (kindly provided by Dr. Janne Lehtiö), MCF-7/LCC2 (kindly provided by Dr. Janne Lehtiö), HEK293 (purchased from ATCC) and COS-7 cells (purchased from ATCC). RPMI-1640 (42401-018, Life Technologies, Grand Island, USA) supplemented with 10% FBS, was used to culture T47D (purchased from ATCC) cells. Phenol red-free DMEM (11880-028, Life Technologies, Grand Island, USA), supplemented with 5% DCC-FCS serum (12676029, Life Technologies, Grand Island, USA) was utilized for all the experiments involving 17β-estradiol (E2) treatment. MCF-7 cells with Flag-BAP (bacterial alkaline phosphatase) or Flag-PAK4 stable expression were cultured in DMEM medium supplemented with 10% FBS and 150 μg/ml G418 (Invitrogen) [25]. All cells were cultured in a humidified incubator at 37°C with 5% CO_2_. Transient transfections of plasmids were performed according to the manufacturer's protocol: 8 μg of total DNA was transfected for each 100-mm cell culture dish (90% cell confluence) using Lipofectamine 2000 (11668-027, Invitrogen, Carlsbad, CA, USA).

### siRNA transfection, PAK4 inhibitor treatment, E2 treatment and tamoxifen treatment

50% confluent cells were transfected with 30 nM small interfering RNA (siRNA) using Lipofectamine RNAiMAX Reagent (13778-150, Invitrogen, Carlsbad, USA) according to the Manufacturer's protocol. The control siRNA (targeting sequences: 5′-TTCTCCGAACGTGTCACG-3′) and the two human PAK4 siRNA (targeting sequences: #1: 5′-AGCTGGTGGCCGTCAAGAA-3′; #2: 5′-CGAGGTG GTAATCATGA-3′) were purchased from GenePharma, Shanghai, China. The cells were harvested at day 3 after transfection. The GNE-2861 compound (Compound 17) was kindly provided by Genentech Inc (South San Francisco, CA, USA) [31]. GNE-2861 was added to the culture medium 24 h before harvest. For the E2 combination treatment, cells were transfected with siRNA for 2 d, and then treated with 10 nM E2 or vehicle for another 24 h. Alternatively, cells were treated with GNE-2861 together with 10 nM E2 or vehicle for 24 h.

### Immunoblotting

Cells were lysed with NP-40 cell lysis buffer (50 mM Tris-HCl pH7.5, 150 mM NaCl, 5 mM MgCl_2_, 1 mM EDTA, 10% Glycerol, 1% NP-40), with freshly added Protease inhibitor (11697498001, Roche, Mannheim, Germany) according to the manufacturer's protocol. 20 μg of cell lysate was loaded into each well for SDS-polyacrylamide gel electrophoresis (PAGE) and was electroblotted onto nitrocellulose membranes (10600008, GE healthcare, Freiburg, Germany). Membranes were incubated with antibodies against β-Actin (A5316) from Sigma (Saint Louis, USA); c-myc (SC40) from Santa Cruz (Dallas, USA); ERα (SC543) from Santa Cruz (Dallas, USA); Flag (F3165) from Sigma (Saint Louis, USA); GFP (MAB2510) from Millipore (Temecula, USA); ubiquitin (FL76) from Santa Cruz (Dallas, USA); PAK4 (total) pAb (6508) generated in our laboratory [25].

### RNA extraction, reverse transcription-PCR (RT-PCR) and quantitative real-time PCR (qPCR) analysis

RNeasy kits were utilized for RNA extraction (74104, Qiagen, Hilden, Germany). RT-PCR was performed using standard procedures, using TaqMan Reverse Transcription Reagents (N808-0234, Applied Biosystems, Foster City, USA). qPCR experimental procedures were previously described [55]. qPCR was performed in a 7500 Fast Real-Time PCR System (Applied Biosystems, Foster City, CA) with FastStart Universal SYBR Green Master mix (04913914001, Roche, Indianapolis, USA) according to conditions specified by the manufacturer. The 36B4 gene was used as an internal control for normalization. Primer sequences for qPCR are shown in more detail (Supplementary Table S1). The specificity of all primer pairs was checked by melting curve analysis.

### Chromatin immunoprecipitation (ChIP) assays and ChIP-Sequencing (ChIP-Seq)

MCF-7 cells were seeded in 150 mm-dishes and grown in phenol red-free DMEM (11880-028, Life Technologies, Grand Island, USA), supplemented with 5% DCC-FCS serum (12676029, Life Technologies, Grand Island, USA). Cells were treated with vehicle or 10 nM E2 for 45 min and chromatin immunoprecipitation (ChIP) was performed as previously described [56], using anti-ERα antibody HC-20 (Santa Cruz, CA) or normal rabbit IgG (Santa Cruz, CA). The immunoprecipitated DNA was assessed by qPCR. Primer sequences used for qPCR are given in more detail (Supplementary Table S1). ChIP-Seq was performed using the Illumina Genome Analyzer following the Manufacturer's protocols. The raw sequencing image data were analyzed by the Illumina analysis pipeline, mapped to the human reference genome (hg19, GRCh37) using Bowtie. Significantly enriched ERα binding regions, comparing to IgG control were identified using the Partek Genomics Suite (Partek^®^ software. Copyright, Partek Inc., St Louis, MO).

### Flow cytometry

EdU (5-ethynyl-2′-deoxyuridine) labeled DNA staining was performed according to the Manufacturer's protocol (C10424, Life Technologies, Eugene, USA) as described in a previous study [57]. 10 μM EdU was added for 1 h before fixation by ice-cold Ethanol (final concentration 70%). The BD LSR II flow cytometer (BD Bioscience) was used to measure the DNA incorporation signal and fluorescence was measured in the FL-4 channel.

### ERE luciferase assays

Cells were co-transfected with ERE/Luci (firefly luciferase) and phRL/CMV (Renilla luciferase) plasmids. Luciferase activity was measured using the Dual-Luciferase Reporter Assay System (E1910, Promega, Mannheim, Germany).

### WST-1 cell proliferation assay

10^4^ cells were seeded into each well of 96-well plates. 1 h before reading the plate, WST-1 reagent (11644807001, Roche, Indianapolis, USA) (1:10 diluted by cell culture medium) was added to each well. The absorbance of the samples was measured against a blank background control using a microplate reader at 450 nm.

### Protein stability assay

Cells were treated with 100 μM cycloheximide (CHX) (C4859, Sigma) for different times or 10 μM MG132 (SC-201270, Santa Cruz) for 8 h. ERα protein levels were analyzed by immunoblotting. Quantification of immunoblotted bands (X-ray film blackening) was performed by ImageJ.

### *In vitro* kinase activity assay

ERα fragments corresponding to amino acid (aa) 1-250; aa 251-420; aa 421-595 and the mutants ERα aa 251-420-S282A; aa 251-420-S294A; aa 251-420-S305A and aa 251-420-T311A were individually expressed as GST-fusion proteins using the bacterial expression vector pGEX-4t-1 (27-1542-01, GE Healthcare, Uppsala, Sweden). GST-fusion proteins were purified using glutathione-Sepharose beads (17-0756-01, GE Healthcare, Uppsala, Sweden) according to the Manufacturer's protocol. His-PAK4 fusion protein was produced as described [26]. *In vitro* protein phosphorylation assays were performed as described [26]. Briefly, ERα phosphorylation was determined in a kinase reaction buffer (50 mM Hepes, pH 7.5, 10 mM MgCl2, 2 mM MnCl2, 0.2 mM dithiothreitol) in the presence of 30 μM ATP and 10 μCi of [γ-^32^P] ATP and in the presence of the kinase (5 μg of His-PAK4) and 5 μg of the substrate (GST, GST-Raf1-aa332-344, or GST-ERα fragments) for 30 min at 30°C. The reaction was stopped by adding sample loading buffer and heating at 95°C for 5 min. Samples were separated by 10% SDS-PAGE and visualized by autoradiography with the PhosphorImager system (Molecular Imager FX, Bio-Rad).

### Ubiquitination assays

HEK-293 cells were transfected with wild-type Flag-ERα or Flag-ERα-S305A. Forty-eight hours after transfection, cells were treated with 10 μM MG132 for 8 h. Ubiquitin was detected by immunoblotting.

### Public datasets

Two publicly available databases were used to explore the prognostic role of PAK4 gene expression in ERα positive clinical samples. The KMplot online tool (http://www.kmplot.com, accessed on April 2015) assembled gene expression data from Affymetrix platforms of different breast cancer cohorts and allows Kaplan-Meier analysis of selected genes [34]. On the KMplot platform, ERα positive patients having received only endocrine therapy (n = 725) or more specifically, only tamoxifen (n = 650) as adjuvant treatment were selected. All annotated Affymetrix probesets with a quality estimated as Jetset best probesets for each of the gene in the A arrays were analyzed [58]. For each member of the PAK family, we used the latest version of the hgu133a.db Bioconductor package (version 3.1.3) as annotation. In case of multiple probesets per gene, the optimal probeset according to the Jetset method was selected for further analysis [58] (Supplementary Table S2). The selected probesets for PAKs are as following: PAK1 209615_s_at; PAK2 208877_at; PAK3 214607_at; PAK4 203154_s_at; PAK5 210721_s_at; PAK6 219461_at. The Metabric gene expression normalized data were downloaded from the European genome-phenome archive platform (http://www.ebi.ac.uk/ega/studies/EGAS00000000083, accessed on March 2015). Metabric used the Illumina HT-12 v3 platform for transcriptional profiling of 1992 breast cancer tumor patients [33]. Clinical variables were obtained from the supplementary material of the original publication [33]. No information was given in the Metabric cohort concerning the type of administered endocrine therapy. 915 ERα positive endocrine therapy treated patients were available in this cohort. The probe for each PAK gene that displays the highest interquartile range was used for the patient outcome analysis (Supplementary Table S3). For two of the PAK genes, more than one probe was available. The ILMN_1712687 probe was selected for PAK2 and the ILMN_1728887 probe was selected for PAK4, since they display the highest interquartile range.

### Statistics

The prognostic role of PAK4 in ERα positive endocrine therapy and tamoxifen treated patients was explored for the Relapse Free Survival (KMplot) and Disease Specific Survival (Metabric) endpoints. Deaths of an unknown cause were excluded from analyses of the Disease Specific Survival endpoint. PAK4 gene expression was dichotomized according to an auto-selected best cut-off (KMplot) or by median-based cut-off (Metabric). The univariable prognostic role of PAK4 (above vs. below the cut-off) was visualized using Kaplan-Meier estimates and compared using log-rank test. All analyses of the Metabric cohort were stratified by site, which is suggested in the original publication [33]. Similar methods were used for all other PAK family genes. The analysis was exploratory and therefore, no multiple testing correction was applied. Spearman correlation analysis was used to calculate the correlation between the expression of the PAK4 and ESR1 genes in the Metabric dataset. Statistical analyses of disease outcome were performed using the KMplot platform and the R statistical software v3.1.0. Two-sample Kolmogorov-Smirnov test (two-sample KS-test) was used for immunoblot quantifications of the CHX experiment. Cox regression analysis and *t*-test were utilized for other statistical analyses. *P* < 0.05 was considered to represent statistical discernibility of differences.

## SUPPLEMENTARY FIGURES AND TABLES


