# Supplementary material for: p21-activated kinase group II small compound inhibitor GNE-2861 perturbs estrogen receptor alpha signaling and restores tamoxifen-sensitivity in breast cancer cells
Source: Oncotarget. 2015 Nov 6;6(41):43853–68. doi: 10.18632/oncotarget.6081 (PMC4791272; doi:10.18632/oncotarget.6081)
Supplement: Supplementary file 1 [file oncotarget-06-43853-s001.pdf]

SUPPLEMENTARY FIGURES AND TABLES

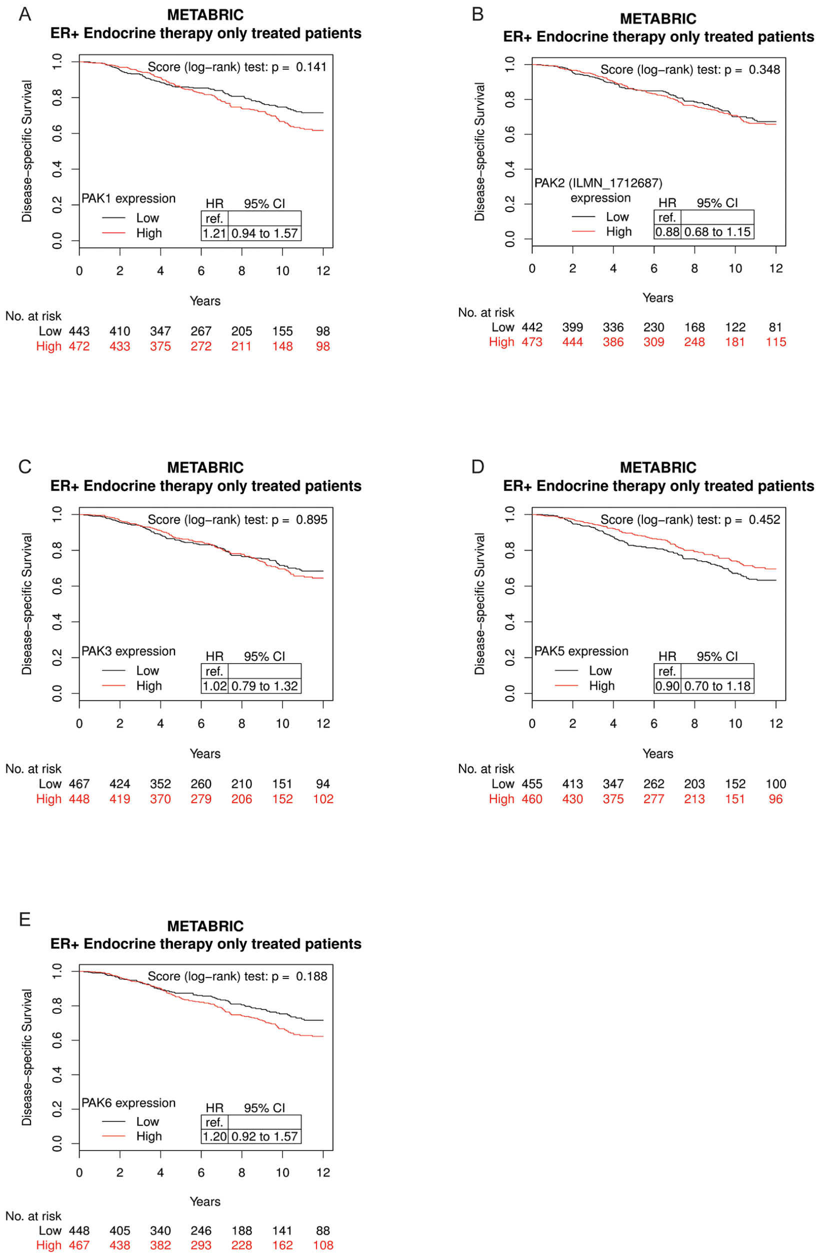

Supplementary Figure S1: The relationship between PAK family member expression levels and endocrine-treated breast cancer patient outcome in the Metabric database. A. Kaplan-Meier plot of disease-specific survival in ER+, endocrine therapy only treated patients in the Metabric database stratified for high (red) and low (black) PAK1 expression levels ($n = 915$; median cut-off; Probe ILMN_1767365: H*R* = 1.21; 95% CI: 0.94–1.57; $P = 0.141$). **B.** Kaplan-Meier plot of disease-specific survival in ER+, endocrine therapy only treated patients in the Metabric database stratified for high (red) and low (black) PAK2 expression levels ($n = 915$; median cut-off; B: Probe ILMN_1712687: H*R* = 0.88; 95% CI: 0.68–1.15; $P = 0.348$). **C.** Kaplan-Meier plot of disease-specific survival in ER+, endocrine therapy only treated patients in the Metabric database stratified for high (red) and low (black) PAK3 expression levels ($n = 915$; median cut-off; Probe ILMN_2130218: H*R* = 1.02; 95% CI: 0.79–1.32; $P = 0.895$). **D.** Kaplan-Meier plot of disease-specific survival in ER+, endocrine therapy only treated patients in the Metabric database stratified for high (red) and low (black) PAK5 (gene named PAK7) expression levels ($n = 915$; median cut-off; Probe ILMN_1770938: H*R* = 0.90; 95% CI: 0.70–1.18; $P = 0.452$). **E.** Kaplan-Meier plot of disease-specific survival in ER+, endocrine therapy only treated patients in the Metabric database stratified for high (red) and low (black) PAK6 expression levels ($n = 915$; median cut-off; Probe ILMN_1708223: H*R* = 1.20; 95% CI: 0.92–1.57; $P = 0.188$).

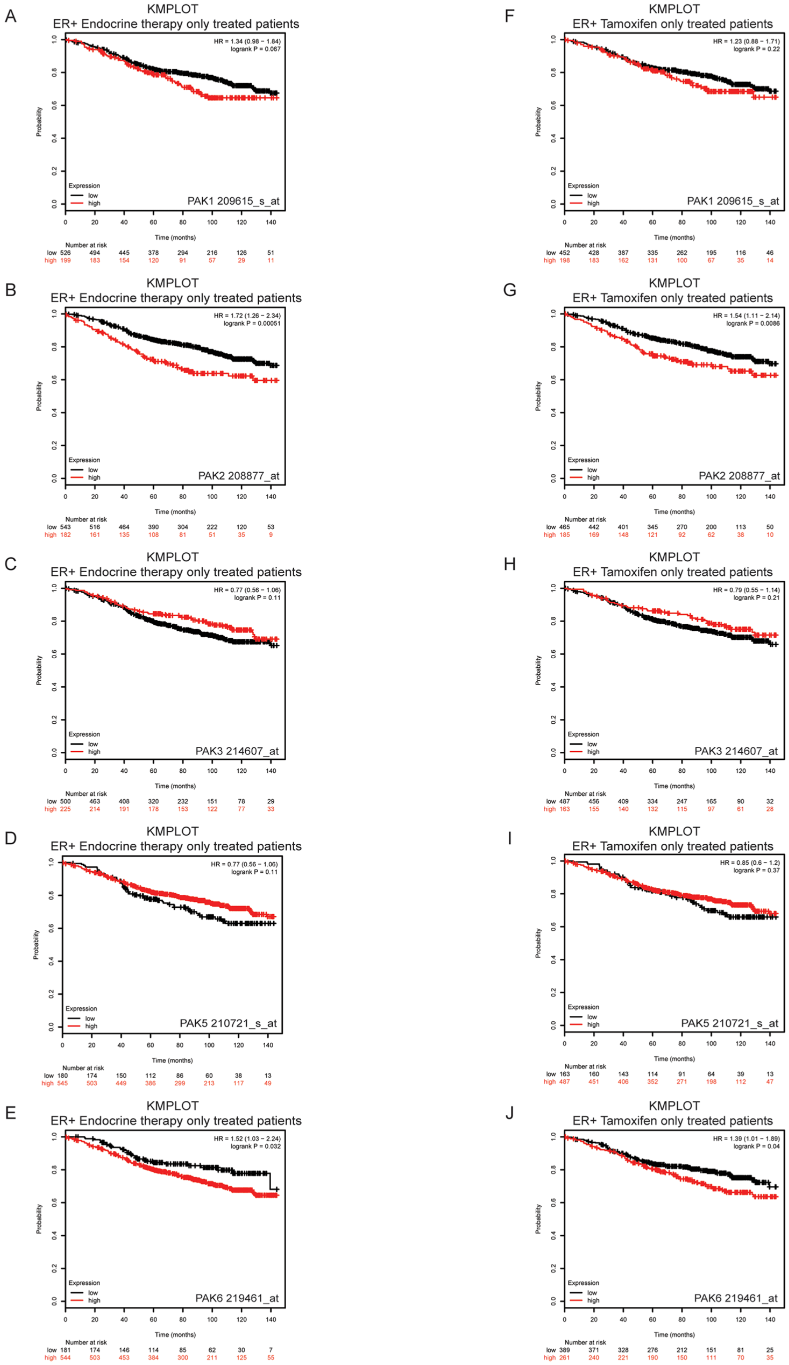

Supplementary Figure S2: The relationship between PAK family member expression levels and endocrine or tamoxifen-only treated breast cancer patient outcome in KMplot database. A–E. Kaplan-Meier plot of disease-specific survival in ER+, endocrine therapy only treated patients in the KMplot database stratified for high (red) and low (black) PAK family member expression levels (n = 725; optimized cut-off; A: PAK1 Probeset 209615_s_at: HR = 1.34; 95% CI: 0.98–1.84; P = 0.067; B: PAK2 Probeset 208877_at: HR = 1.72; 95% CI: 1.26–2.34; P = 0.00051; C: PAK3 Probeset 214607_at: HR = 0.77; 95% CI: 0.56–1.06; P = 0.11; D: PAK5 (gene named PAK7) Probeset 210721_s_at: HR = 0.77; 95% CI: 0.56–1.06; P = 0.11; E: PAK6 Probeset 219461_at: HR = 1.52; 95% CI: 1.03–2.24; P = 0.032). **F–J.** Kaplan-Meier plot of disease-specific survival in ER+, tamoxifen-only treated patients in the KMplot database stratified for high (red) and low (black) PAK family member expression levels (n = 650; optimized cut-off; F: PAK1 Probeset 209615_s_at: HR = 1.23; 95% CI: 0.88–1.71; P = 0.22; G: PAK2 Probeset 208877_at: HR = 1.54; 95% CI: 1.11–2.14; P = 0.0086; H: PAK3 Probeset 214607_at: HR = 0.79; 95% CI: 0.55–1.14; P = 0.21; I: PAK5 (gene named PAK7) Probeset 210721_s_at: HR = 0.85; 95% CI: 0.6–1.2; P = 0.37; J: PAK6 Probeset 219461_at: HR = 1.39; 95% CI: 1.01–1.89; P = 0.04).

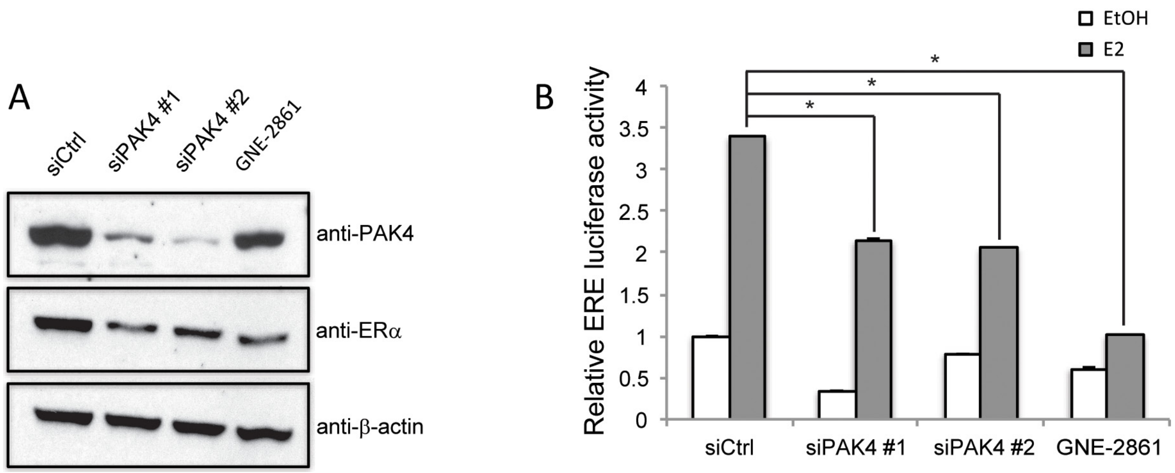

Supplementary Figure S3: PAK4 inhibition impairs ERα signaling in T47D cells. A. PAK4 depletion or functional inhibition of group II PAKs reduces ERα protein levels in T47D cells. T47D ERα positive human breast cancer cells were transfected with siControl or siPAK4 oligos (#1 or #2) for 72 h, or treated with 50 µM GNE-2861 for 24 h. ERα, PAK4 and β-actin levels were determined by immunoblot. **B.** PAK4 depletion or functional group II PAK inhibition reduces the activity of estrogen receptor-induced signal transduction in T47D. T47D cells were transfected with siControl, siPAK4 oligos (#1 or #2), or treated with GNE-2861 as described in A. 24 h before measurement, cells were transfected with an ERE luciferase reporter. After 18 h, cells were treated with 10 nM E2 or vehicle, and an ERE-luc luciferase assay was carried out 6 h after E2 addition. Shown values represent mean ± s.d. (n = 3), which is representative for three independent experiments. * - $P < 0.05$ for Flag-PAK4 group versus control, according to *t*-test.

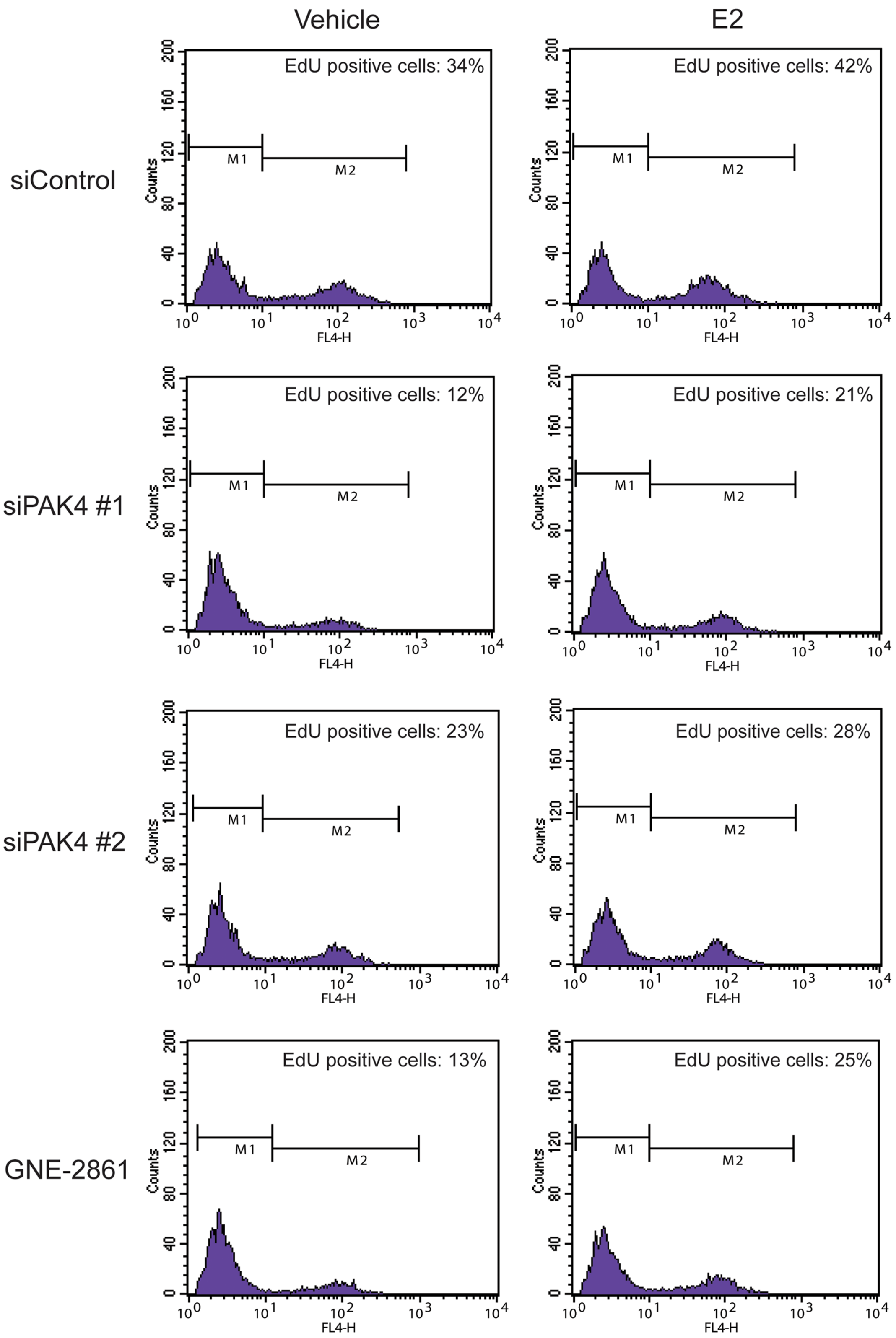

Supplementary Figure S4: PAK4 depletion or functional group II PAK inhibition impairs cell proliferation in MCF-7 cells. MCF-7 cells were transfected with siControl, siPAK4 oligos (#1 or #2), or treated with GNE-2861. Cells were then treated with 10 nM E2 or vehicle for 6 h before fixation. EdU was added at a concentration of 10 µM during the last 1 h. Flow cytometry histograms shows negative (M1) and EdU-positive (M2) cell populations and the fraction of EdU-positive cells is indicated.

Supplementary Table S1: qPCR primers used in this study. Column 1 contains the primer name and the information whether the primer is a forward or reverse primer. Column 2 contains the oligonucleotide sequence in 5′ to 3′ direction. Column 3 specifies in which figure the results related to this primer are displayed.

Primer name	Sequence	Figure
Neg Ctrl #1 Forward	TCTCTTCCTCAGCCCCTTTGT	3D
Neg Ctrl #1 Reverse	GTACCAAGCACAGAGCAAATGG	3D
Neg Ctrl #2 Forward	CACCATGTTTGGGAAGAGGAA	3D
Neg Ctrl #2 Reverse	GCGGTGCTCGAAGTTGGA	3D
Binding site #1 Forward	CAGGTCATGGGTACGGAAGTG	3D
Binding site #1 Reverse	CGACCATTCCGAGGTCATTC	3D
Binding site #2 Forward	GGTGAGGAGACGGCTTTAGCT	3D
Binding site #2 Reverse	TCTGATTGTATCCTCTCCTGCTAAAA	3D
36B4 Forward	GTGTTCGACAATGGCAGCAT	4C
36B4 Reverse	GACACCCTCCAGGAAGCGA	4C
PAK4 Forward	ATGTGGTGGAGATGTACAACAGCTA	4C
PAK4 Reverse	GTTCATCCTGGTGTGGGTGAC	4C
ERα Forward	GCTACGAAGTGGGAATGATGAAAG	4C
ERα Reverse	TCTGGCGCTTGTGTTTCAAC	4C
ADORA1 Forward	TTCCACACCTGCCTCATGGT	4C
ADORA1 Reverse	GCGGTCCACAGCAATTGC	4C
Cyclin D1 Forward	CACGCGCAGACCTTCGT	4C
Cyclin D1 Reverse	GGGCGGATTGGAAATGAAC	4C
EGR3 Forward	GCCCATTACAATCAGATGGCT	4C
EGR3 Reverse	GCTCGAATAAGAGAGTTCCGGA	4C
GREB1 Forward	ATCAGCTGCTCGGACTTGCTG	4C
GREB1 Reverse	TGAGCTCCGGTCCTGACAGATG	4C
IL-20 Forward	CGATGCTGCCTCCTGCG	4C
IL-20 Reverse	GCTGCCTGAGGTTCCAGCTT	4C
PDZK1 Forward	GCCAGGCTCATTCATCAAAGA	4C
PDZK1 Reverse	CCTCTAGCCCAGCCAAGTCA	4C
PKIB Forward	GAGGCTCTCTCCGTGAAGGA	4C
PKIB Reverse	TCCAATTGGTCTTGTGTTGTTTTT	4C
PS2 Forward	CATCGACGTCCCTCCAGAAGAG	4C
PS2 Reverse	CTCTGGGACTAATCACCGTGCTG	4C

Supplementary Table S2: KMplot probeset selection for PAK family members. Column 1 contains probeset IDs. Column 2 contains probeset gene symbol and the Jetset status of the gene according to KMplot. Green probeset means it is the Jetset best probeset for a gene in the A arrays; Red probeset means it is not the Jetset best probe set for a gene in the B array. Column 3 contains probeset annotation in KMplot. For PAK2, PAK3 and PAK5, other names (synonyms) are also used in KMplot. The PAK6 probeset is also annotated as PAK5 probeset in KMplot. Column 4 contains probeset gene symbol according to the newest version of the hgu133a.db Bioconductor package (annotation for array A version 3.1.3). In column 1, the probeset used in this study for each PAK family member is highlighted in bold. The probeset selection was based on the correct gene annotation in the hgu133a.db Bioconductor package and the qualification as a KMplot green probeset.

Probeset ID	Kmplot	Annotation by Kmplot	Annotation by hgu133a.db
202161_at	PAK1 Green probeset		PKN1
**209615_s_at**	PAK1 Green probeset		PAK1
205962_at	PAK2 Red probeset		PAK2
208875_s_at	PAK2 Red probeset		PAK2
208876_s_at	PAK2 Red probeset		PAK2
**208877_at**	PAK2 Green probeset	PAK2, also named PAK67 or PAKgamma	PAK2
208878_s_at	PAK2 Red probeset		PAK2
202547_s_at	PAK3 Red probeset		ARHGEF7
202548_s_at	PAK3 Green probeset		ARHGEF7
**214607_at**	PAK3 Green probeset	PAK3, also named hPAK3, bPAK, MRX30 or MRX47	PAK3
**203154_s_at**	PAK4 Green probeset		PAK4
215326_at	PAK4 Red probeset		PAK4
33814_at	PAK4 Red probeset		PAK4
**210721_s_at**	PAK7 Green probeset	PAK5, also named PAK7, KIAA1264	PAK7
213990_s_at	PAK7 Red probeset		PAK7
**219461_at**	PAK6 Green probeset	also annotated as PAK5 probeset in Kmplot	PAK6

Supplementary Table S3: Metabric probe selection for PAK family members. Column 1 contains illumina probe IDs. Column 2 contains probe annotation in the Metabric database. Column 3 contains probeset interquartile range in the Metabric dataset. In column 1, for each PAK family member gene, the probe displaying the highest interquartile range was chosen for analysis and is highlighted in bold.

Illumina Probe ID	Annotation	Interquartile range
**ILMN_1767365**	PAK1	0.74
ILMN_1676385	PAK2	0.47
**ILMN_1712687**	PAK2	0.49
**ILMN_2130218**	PAK3	0.18
**ILMN_1728887**	PAK4	0.57
ILMN_1763187	PAK4	0.20
ILMN_2354673	PAK4	0.35
**ILMN_1708223**	PAK6	0.77
**ILMN_1770938**	PAK7 (also named PAK5)	0.20
